# Discovery of Potential Antileishmanial Compounds Through Phenotypic Screening of an Alkaloid Library

**DOI:** 10.3390/molecules30214210

**Published:** 2025-10-28

**Authors:** Cathy Soh-Kamdjo, María-Cristina González-Montero, Carlos García-Estrada, Estela Melcón-Fernández, Celia Fernández-Rubio, Yolanda Pérez-Pertejo, Rosa M. Reguera, Rafael Balaña-Fouce

**Affiliations:** 1 Departamento de Ciencias Biomédicas, Facultad de Veterinaria, Universidad de León, Campus de Vegazana s/n, 24007 León, Spain; csohk@unileon.es (C.S.-K.); magom@unileon.es (M.-C.G.-M.); cgare@unileon.es (C.G.-E.); cferrb@unileon.es (C.F.-R.); myperp@unileon.es (Y.P.-P.); rmregt@unileon.es (R.M.R.); 2 Instituto de Biomedicina (IBIOMED), Universidad de León, Campus de Vegazana s/n, 24007 León, Spain

**Keywords:** antileishmanial alkaloids, phenotypic high-throughput screening, chemoinformatics

## Abstract

Visceral leishmaniasis caused by *Leishmania donovani* is one of the major neglected tropical diseases attributable to parasitic protozoa. In the absence of an effective vaccine, chemotherapy remains the only available therapeutic option. However, current treatments rely on a limited number of drugs that are largely obsolete, highly toxic or require intravenous administration, and their extensive use has led to the emergence of drug resistance. Consequently, the discovery of new antileishmanial agents is an urgent priority. In this study, a commercial library of 449 alkaloids in a high-throughput screening format was evaluated against both axenic bone marrow-derived amastigotes and intramacrophagic amastigotes from mice infected with *L. donovani* IRFP, a strain engineered to emit infrared fluorescence in its viable form. Six isoquinoline-type alkaloids showed the best antileishmanial efficacy against intramacrophagic amastigotes while exhibiting minimal cytotoxicity toward RAW 264.7 and HepG2 cell lines, with a promising selective index higher than four, and good mouse intestinal tolerance in mouse organoids. Among these compounds, the protoberberine scaffold emerged as the most promising candidate for further drug development.

## 1. Introduction

Neglected tropical diseases (NTDs) comprise twenty primarily infectious and parasitic diseases that disproportionately affect populations in low- and middle-income countries. These diseases can be fatal and not only impose significant health problems but also impede economic and social development in endemic regions due to enormous health costs and loss of workforce productivity among adults and education disruption among children. NTDs affect nearly one-sixth of the global population, with Africa bearing the greatest health, economic and social burden [1]. In response to this challenge, the African Union and Uniting to Combat NTDs adopted a declaration at the Kigali Summit on Malaria and NTDs in March 2022, aiming to reduce NTD infections by 90% and eliminate at least one NTD in 100 countries by 2030, in alignment with the WHO roadmap [2]. However, NTD control remains difficult due to limited or absent availability of vaccines, outdated drug treatments and the emergence of drug resistance, underscoring the urgent need for novel therapeutic interventions [3].

Visceral leishmaniasis (VL) is an important vector-borne protozoan NTD that remains a serious public health concern, particularly in sub-Saharan Africa, where the highest prevalence and mortality rates are reported [4,5]. According to the 2023 WHO report on VL, 40% of the 200 reporting countries were classified as endemic, collectively reporting over 11,000 new cases [6]. In the absence of an effective vaccine, current VL treatment relies on a limited number of drugs, including pentavalent antimonials, amphotericin B, miltefosine and paromomycin. Most of these drugs are outdated and associated with significant toxicity, complex administration requirements, and, in some cases, chemical instability at the point of care [7]. These limitations have led to reduced treatment efficacy and the emergence of drug-resistant strains. In recent years, no new therapeutic alternatives have been developed, apart from combinations of existing drugs, which have shown acceptable results [5].

In the urgent search for new active compounds to address this global health challenge, two main strategies have been employed in drug discovery: (i) phenotypic screening based on antileishmanial activity, and (ii) target-based approaches that rely on the identification of specific pharmacological targets [8,9]. Both strategies have been adapted to high-throughput screening (HTS) platforms, enabling large-scale evaluation of chemical libraries. In addition, computational approaches—traditionally considered complementary tools—are now playing an increasingly role in drug discovery workflows by optimizing resource allocation, reducing costs and timelines, and improving overall outcomes [10,11].

Natural plant-based products have long been recognized as an important source of secondary metabolites with diverse pharmacological activities [12]. Among these, alkaloids—nitrogen-containing heterocyclic compounds biosynthesized from amino acids and distributed across various botanical families—have demonstrated a wide range of bioactivities, including antiproliferative (antitumor), antibacterial, antiparasitic, anticoagulant and antioxidant properties. These characteristics make them promise candidates for drug development [13]. For decades, alkaloids have served as chemical templates for the design of numerous synthetic and semi-synthetic derivatives, many of which display potent biological activity both in vitro and in vivo [14,15,16].

The objective of the present study was to identify new antileishmanial drug candidates by screening a commercial library of plant-derived alkaloids using a platform based on axenic amastigotes of *L. donovani* IRFP—the causative agent of VL—genetically modified to emit infrared fluorescence during their viable stage (hereafter referred to as *L. donovani* IRFP) [17]. To this end, single-dose assays at 10 μM and 1 μM, as well as dose–response assays, were performed. Compounds showing significant activity in the initial screening were subsequently clustered according to chemical structure using the DataWarrior tool [18], and representative compounds were further evaluated in dose response assays against axenic amastigotes. The efficacy of the most potent compounds was subsequently assessed in ex vivo intramacrophagic amastigotes isolated from mice infected with *L. donovani* IRFP. In parallel, cytotoxicity of the most active compounds was evaluated in RAW 264.7 and HepG2 cell lines, as well as in mouse intestinal organoids. Finally, chemoinformatic analysis was conducted to assess the most favorable structural features of the most effective and safest alkaloids.

## 2. Results

### 2.1. Strategy of Compound Screening Against Leishmania

**Figure 1:**
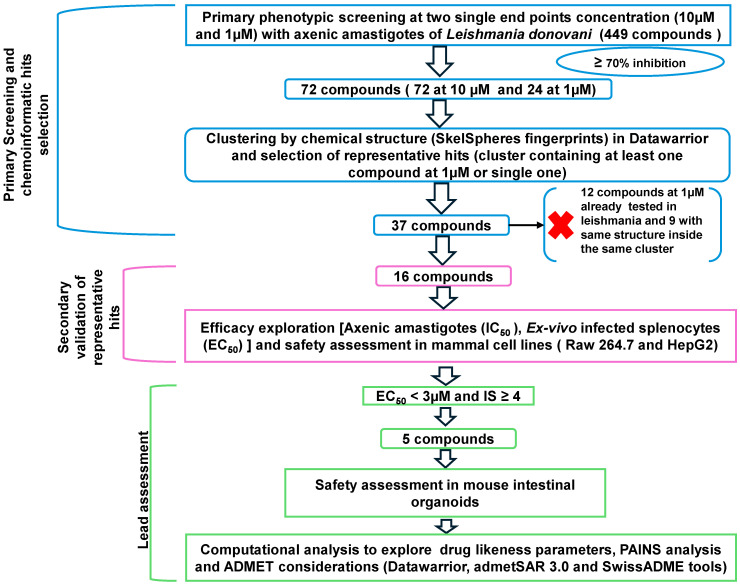
A stepwise screening cascade was applied to 449 alkaloids from the MedChemExpress library (MedChemExpress, Sollentuna, Sweden. Ref.: HY−L071). Compounds were first tested in single-shot experiments at 10 μM and 1 μM, identifying a total of 72 and 24 hits, respectively, using a ≥70% inhibition cut-off. Chemoinformatic clustering by structural similarity reduced the set to 16 representative compounds. These were validated through dose/response assays on axenic amastigotes and ex-vivo infected splenic explants, followed by cytotoxicity testing in mammalian cells to determine safety and Selectivity Index (SI). Each open arrowhead represents subsequent steps in the screening process. A black arrow and a red cross indicate that those compounds were not further studied.

Non-target-based platforms of drug discovery (phenotypic screening platforms) are generally more relevant than those based on specific targets, as they can avoid a high rate of false positives. In this regard, some good practices have been recommended for designing reproducible phenotypic platforms for the detection and prioritization of results [19]. The strategy followed in this work to identify compounds effective against *L. donovani* IRFP from the commercial collection of alkaloid drugs (MedChemExpress, Sollentuna, Sweden. Ref.: HY−L071), is shown in Figure 1. In a first step, two single concentrations (10 µM and 1 µM) were tested in 96-well plates with 25,000 axenic amastigotes [20] in two independent experiments to individually evaluate the activity of these compounds. The percentage of inhibition at 72 h was determined using a cut-off value of ≥70% growth inhibition in at least one of the two experiments to classify the compound as active or inactive. Amphotericin B at 10 µM was used as the positive control, and 0.1% DMSO as the negative control. Plates with a Z’ factor ≥ 0.5 were considered valid, according to the formula described by Zhang and co-workers [21] (Figure S1, Supplementary Material).

### 2.2. Primary Screening and Cheminformatics Hit Selection

**Figure 2:**
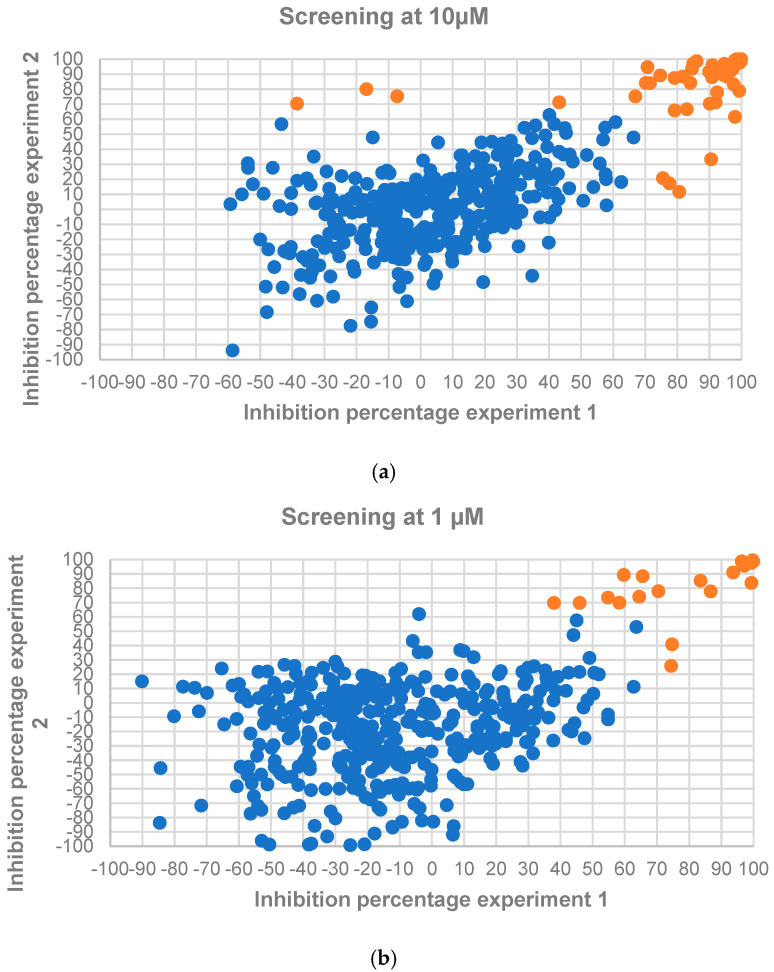
*L. donovani* IRFP axenic amastigote growth inhibition caused by the 449 employed screening compounds from the Alkaloids Library (MedChemExpress, Sollentuna, Sweden. Ref.: HY−L071) at 10 µM (**a**) and 1 µM (**b**). Orange dots represent compounds surpassing the established cut-off threshold of ≥ 70% inhibition in at least one of the experimental replicates, while blue dots represent those molecules not passing the threshold.

The 449 compounds in the Alkaloids Compound Library (MedChemExpress, Sollentuna, Sweden. Ref.: HY−L071) were initially screened at single concentrations of 10 µM and 1 µM in two independent assays against *L. donovani* IRFP. Seventy-two and 24 compounds—corresponding to 16.04% and 5.35% of the collection, respectively—met the previously established cut-off value of 70% growth inhibition in at least one of the experimental replicates at 10 µM and 1 µM (Figure 2a,b). A list of active compounds identified in these two primary screens is provided in Table S1 (Supplementary Material).

**Figure 3:**
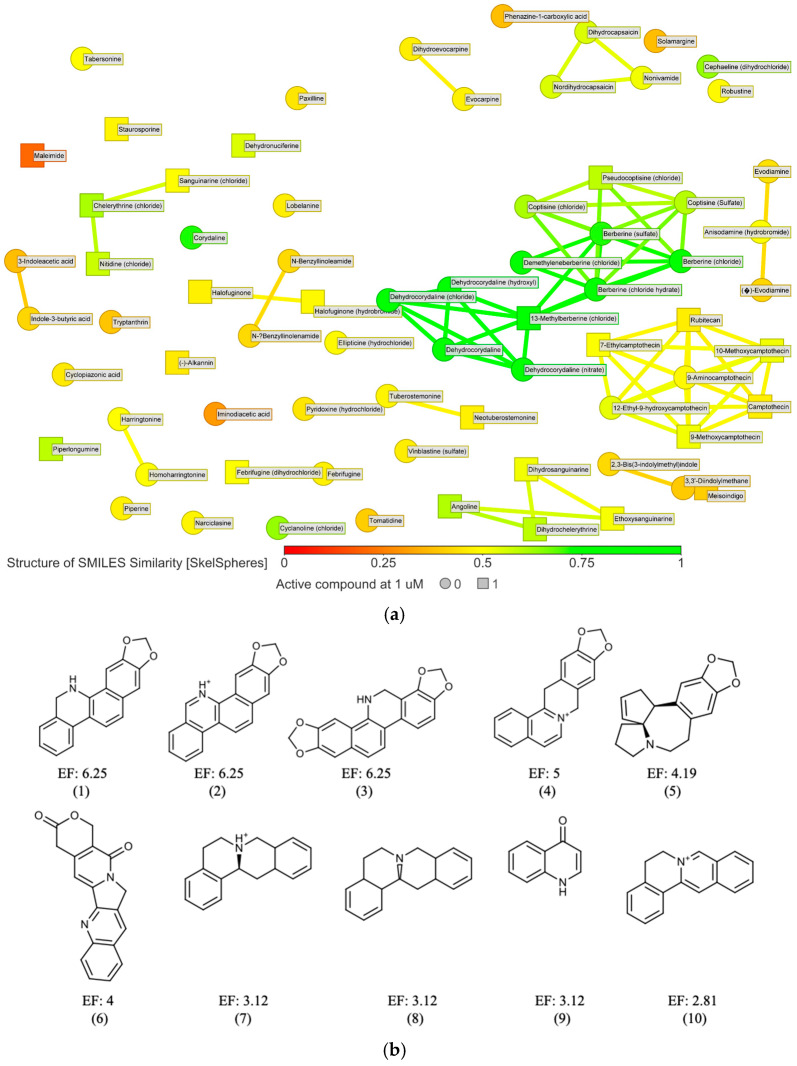
(**a**) 2D similarity chart of the 72 active compounds at 10 µM from the phenotypic screening. Markers are dynamically colored based on structural similarity to the selected reference compound dehydrocorydaline. Similar neighbors are connected by lines. Squares indicate compounds active at 1 µM, while circles represent those that were not; (**b**) Murcko scaffolds were generated using the criteria described in Section 4. Only those with an enrichment factor (EF) ≥ 2 and containing at least two unique active molecules (excluding duplicates due to different salt forms) were included (represented by numbers from 1 to 10 below each scaffold). Total scaffolds found are listed in Table S2 (Supplementary Material).

The 72 active compounds at 10 µM were grouped using the DataWarrior tool based on their chemical structure (SkelSpheres fingerprint), to guide the selection of some representative results from some clusters containing at least one active hit at 1 µM (Figure 3a and Table S1). From this step, we selected compounds belonging to clusters that contained at least one active or isolated compound at 1 µM, resulting in a total of 37 compounds. Next, the alkaloids derived from camptothecin (7 molecules) were discarded, as our group had described the antileishmanial effect of these compounds in previous studies [22]. We also excluded an additional 7 compounds showing activity at 1 µM, since their antileishmanial effect had already been described by other authors. Finally, for those compounds whose active ingredient was present in multiple salt forms, only one was selected for analysis, typically the chloride salt.

The DataWarrior tool was used to filter, group, and prioritize compounds based on their chemical structures [18] (Figure 3a and Figure S2). A subsequent analysis of the basic molecular scaffolds of all 449 compounds of the library using the Murcko framework algorithm (Figure 3b) revealed a series of privileged pharmacophores among the active molecules [23]. These were classified into different groups, notably including compounds containing the isoquinoline ring (such as benzo[c]phenanthridines and proto-berberines), as well as derivatives of stemonin and indurubin (Table S2 in Supplementary Material).

Subsequently, compounds with EC_50_ values below 3 μM against intramacrophagic amastigotes were evaluated for adequate safety margin, as determined by the selectivity index (SI). To this end, we calculated SIs from 16 preselected results from the ex vivo system. With this aim, dose–response curves were performed in duplicate across at least three independent experiments using RAW 264.7 mouse macrophages (as a model of host cells harboring amastigotes) and HepG2 (as a model for assessing systemic toxicity). The results are shown in Table 1, and the corresponding dose–response curves are represented in Figure S3 (Supplementary Material).

**Figure 4:**
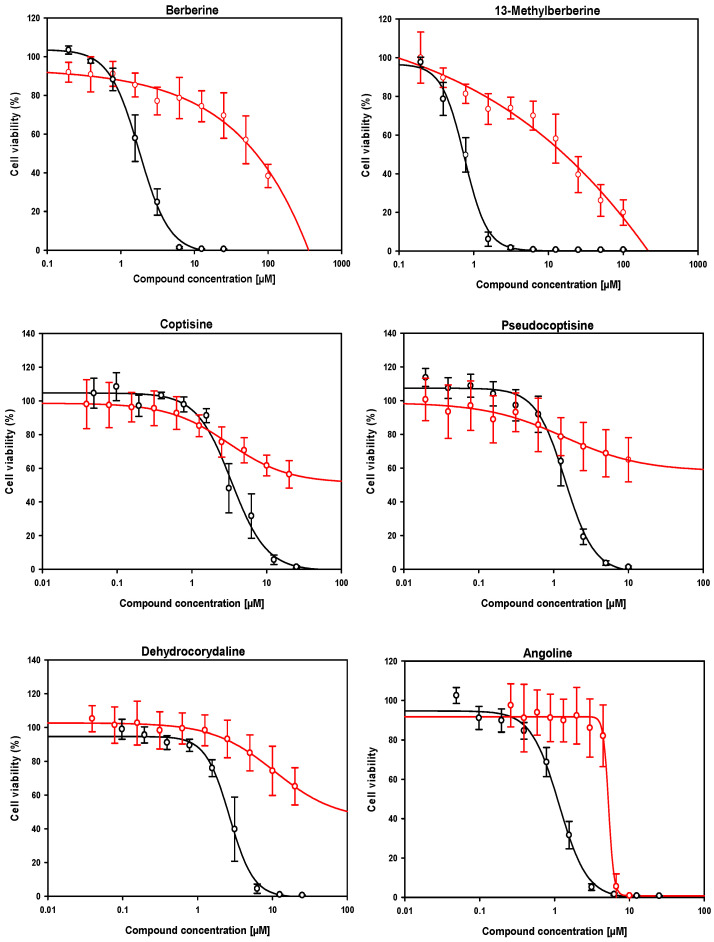
Dose–response curves of the six compounds meeting the lead criteria of EC_50_ < 3 µM and SI ≥ 4, identified from the Alkaloids Compound Library (MedChemExpress, Sollentuna, Sweden. Ref.: HY−L071). The effect on intramacrophagic amastigotes in primary cultures of infected mouse splenocytes and the cytotoxic effect in RAW 264.7 mouse macrophages are shown in black and red, respectively. EC_50_ and CC_50_ values, listed in Table 1, were calculated using the SigmaPlot^TM^ statistical software version 10.0. Each point represents the mean ± SD of at least three different experiments performed in triplicate.

With a few exceptions (dehydrocorydaline, coptisine and, to a lesser extent, 13-methylberberine), the compounds exhibited significantly higher antileishmanial activity against axenic amastigotes than against intramacrophagic amastigotes. This affected the value of the SI, which was lower when the CC_50_ values were compared to the IC_50_ values obtained in axenic amastigotes versus intramacrophagic amastigotes, likely reflecting differences in compound uptake. Some compounds exhibited unacceptable EC_50_ values, close to or exciding 10 μM, including dihydrochelerythrine, dihydrosanguinarine, neotuberostemonine, tuberostemomine, dehydronuciferine and meisoindigo. Other compounds were toxic or extremely toxic to cells, with SI < 4, including sanguinarine and nitidine. Consequently, we selected a group of six compounds, namely angoline, 13-methylberberine, pseudocoptisine, dehydrocorydaline and coptisine (despite its EC_50_ value being slightly higher than threshold), that met the dual criteria of EC_50_ < 3 μM and SI ≥ 4, and whose dose–response curves are shown in Figure 4. Additionally, berberine was included to compare its effects with those of its analogue, 13-methylberberine.

Interestingly, the tolerance of these compounds for oral administration was good as demonstrated using mouse intestinal organoids (see Figure S4 in Supplementary Material). With the exception of angoline, which showed significant toxicity, the remaining compounds exhibited low toxicity, thus suggesting that future formulations may be feasible without major safety concerns.

Of the six selected compounds, we explored druglikeness and ADMET properties using three different computational chemistry tools. All three tools consistently indicated that the compounds comply with Lipinski’s rules [24]. Furthermore, SwissADME showed that the compounds also passed other druglikeness filters. No alerts related to the PAINS filter were observed across the three tools. Key pharmacokinetic parameters are summarized in Table 2 (other druglikeness parameters are listed in Table S3).

## 3. Discussion

The need for drugs to treat visceral leishmaniasis is one of the key priorities in the WHO’s objectives, highlighting the outdated nature of current treatments and the lack of new chemical entities with good efficacy and safety profiles. Given that the compounds identified so far as leads and second-line drug candidates are still far from clinical approval, the only option currently validated by DNDi is the combination of known drugs, which has shown acceptable results to date [25]. Urgent research is therefore needed to identify chemical compounds with antileishmanial activity from diverse sources, to expand the pool of candidates for further preclinical and clinical studies. Plants are a rich source of natural products and plant-derived alkaloids have played a significant role over the past few decades in drug discovery. In fact, their multiple antitumor, antimicrobial, and anti-inflammatory properties, among others, suggest that these compounds hold great promise for the development of new drugs with antileishmanial properties [13].

Of the 16 alkaloids identified as having strong antileishmanial effect at an IC_50_ < 3 μM against axenic amastigotes of *L. donovani* IRFP, 11 share the well-characterized benzo[c]phenanthridine structure. This pharmacophore consisting of a four-fused-ring structure that includes an isoquinoline group, has been described as a promising antiparasitic scaffold [26], although not all their members have acceptable selective toxicity. Within this group, only angoline and, to a lesser extent chelerythrine, met the lead compound criteria of EC_50_ < 3 μM in intramacrophagic amastigotes and a SI ≥ 4 (chelerythrine barely had a SI = 3.4). These results are consistent with those reported for the orally administered antileishmanial drug miltefosine (IC_50_ for axenic amastigotes ranging from 0.4 to 3.8 µM), although they are higher than those observed for amphotericin B (0.6 to 0.7 µM), which has the drawback of requiring intravenous administration [27]. The rest of the compounds in this cluster either failed due to toxicity issues (ethoxysanguinarine, sanguinarine, and nitidine) or exhibited a significant loss of antileishmanial activity against intramacrophagic amastigotes (dihydrochelerytrine and dihydrosanguinarine), likely due to the necessity of crossing multiple permeability barriers to reach the parasite [28]. Benzo[c]phenantridines are known to be cytotoxic agents for cancer cells, and some of them have been shown to induce apoptosis. The mechanism of action of these compounds may be attributed to their flat structure, as they are considered intercalating agents capable of interfering with the activity of type I and II DNA-topoisomerases [29,30]. However, these effects have been poorly studied in trypanosomatids [31]. Castillo and co-workers [32] demonstrated that chelerythrine and nitidine exhibited in vitro IC_50_ values very similar to those of glucantime against axenic amastigotes of *L. amazonensis*, although their antileishmanial effect in vivo was moderate. Induction of apoptosis like death by inhibition of Protein Phosphatase 2C (PP2C) has been proposed as potential mechanism of action of these compounds [33].

Other alkaloids also derived from the isoquinoline heterocycle and featuring a four-ring fused system, were proto-berberine derivatives, such as berberine, 13-methylberberine, pseudocoptisine, and dehydrocorydaline. All of these compounds met the established criteria for efficacy against *L. donovani* IRFP intramacrophagic amastigotes and selectivity in cell cultures. Among the compounds evaluated, coptisin showed a slight reduction in efficacy against intramacrophagic amastigotes, with a moderate increase in EC_50_ of 3.39 µM. However, its favorable SI justified its consideration as a possible lead, especially given the lack of existing literature on its antileishmanial activity. The mechanism of action of berberine has been linked to the depolarization of the mitochondrial membrane of the parasite, causing cell arrest [34]. In addition, berberine chloride triggers apoptosis-like death following increased generation of reactive oxygen species [35] and induces immunomodulatory processes in the host [36]. However, to the best of our knowledge, no data regarding the potential antileishmanial effect of the remaining proto-berberine compounds is currently available, although several molecular targets have been suggested [37].

Two derivatives of stemonin, an alkaloid from traditional Chinese medicine with anti-inflammatory properties [38] and containing the characteristic pyrrole-[1,2-α]azepine scaffold, namely tuberostemonin and neotuberostemonin, were identified on our screening platform. Despite showing interesting effects against axenic amastigotes, they lost their antiparasitic activity against intramacrophagic amastigotes. Similar results were obtained with the indole alkaloid meisoindigo, a compound currently under investigation for its antitumor efficacy against leukemia [39], with no prior reports on antileishmanial activity. According to the results shown in Table 1, mesoindigo exhibited a marked loss of antileishmanial activity in the ex vivo model of intramacrophagic amastigotes and showed high cytotoxicity. Accordingly, we decided not to pursue further research on this compound.

It is worth highlighting a group of seven alkaloids derived from the quinoline pharmacophore isolated from the Chinese tree *Camptotheca acuminata.* These compounds, containing five fused pyrano indolizino quinoline rings, are exemplified by camptothecin, the benchmark molecule of this class. These compounds were identified in the primary screenings at both 1 μM and 10 μM concentrations but were subsequently discarded because of their toxicity. Camptothecin derivatives are well-known anti-tumor agents, and some semisynthetic derivatives, such as topotecan and irinotecan, are currently used to treat different types of cancer [40]. All these compounds irreversibly target DNA topoisomerase I, a nuclear enzyme responsible for unwinding DNA prior to gene transcription and DNA replication [41], and were previously tested by our group, showing interesting antileishmanial efficacy in vitro [22]. DNA topoisomerase I was identified as an important druggable target against trypanosomatids (including *Leishmania*) due to its peculiar structure as a heterodimeric enzyme (unlike the monomeric conformation characteristic of the other species) [42]. Nevertheless, due to the high cytotoxicity of this type of compound, further studies on this cluster were discontinued.

After evaluating efficacy and safety, we retained six compounds that met acceptable criteria for predictive pharmacokinetic analysis. Angoline, pseudocoptisine, coptisine, dehydrocorydaline, and 13-methylberberine, the analogue of berberine, were evaluated in silico to predict their ADMET profile. All six compounds complied with Lipinski’s rules according to the two applications described in Section 4, suggesting good gastrointestinal absorption. Furthermore, our in vitro results on the tolerability of these compounds in mouse intestinal organoids showed that none of them pose toxicological concerns, supporting their suitability for oral administration in future formulations. Angoline demonstrated a high predicted potential to inhibit key cytochrome P450 isoforms (CYP1A2, CYP2C9, CYP2D6, CYP2C19, and CYP3A4). However, CYP3A4, the most abundant isoform in the liver and intestine of adults and responsible for the metabolism of approximately 50% of small molecules [43], showed a predicted inhibition probability below 0.4 for the remaining compounds, suggesting that their potential for clinically relevant interactions may be low. Unfortunately, the low tolerance observed in mouse organoids and the bad toxicological predictions from both tools revealed that angoline has a high probability of mutagenicity and carcinogenicity, which precludes its further development. Nevertheless, considering all evaluated parameters, dehydrocorydaline was selected as the lead compound from the initial set of six candidates.

## 4. Materials and Methods

### 4.1. Preparation of the Drug Library

The alkaloids library (Ref.: HY−L071), purchased from MedChemExpress (MedChemExpress, Sollentuna, Sweden) in 2023, contains 449 structurally diverse molecules, including indoles, quinolines, isoquinolines, pyrrolidines, pyridines, pyrrolizidines, tropanes, terpenoids and steroids. Most compounds were dissolved in DMSO at a stock concentration of 10 mM and stored at −80 °C. Intermediate dilution plates were prepared at the required concentrations, stored at −20 °C, and subsequently used for the main parasite screening assays (Table S4).

### 4.2. Experimental Animals and Ethical Statement

To obtain bone marrow axenic amastigotes and/or intramacrophagic amastigotes, six- to eight-week-old female Balb/c mice were purchased from Janvier Labs (St Berthevin Cedex, France). All animal procedures were conducted in accordance with the Spanish legislation (RD 53/2013) and the European Union Directive (2010/63/UE). All protocols were approved by the Junta de Castilla y Leon (authorization number OEBA 010-2023).

### 4.3. Leishmania donovani Strain

The *L. donovani* IRFP strain was previously engineered in our laboratory [17] to constitutively express the *iRFP* gene, which encodes the infrared bacteriohytochrome protein from *Rhodopseudomonas palustris* [44]. The strain was maintained as free-living promastigotes in Schneider’s insect medium (Sigma-Aldrich, Merck, Darmstadt, Germany) supplemented with 20% (*v*/*v*) fetal bovine serum (FBS; Gibco, ThermoFisher Scientific, Waltham, MA, USA) and an antibiotic mixture containing 10,000 units/mL penicillin and 10,000 µg/mL streptomycin (Hyclone^TM^, ThermoFisher Scientific, Waltham, MA, USA). Cultures were incubated at 26 °C following previously described conditions [45].

### 4.4. Isolation of Axenic Amastigotes of L. donovani IRFP from Bone Marrow

Female Balb/c mice aged from 6 to 8 weeks were intraperitoneally inoculated with 1.5 × 10^9^ infective *L. donovani* IRFP metacyclic promastigotes. Between 8 and 12 weeks after infection, mice were humanely euthanised and bone marrow was collected from the femurs and tibias of both hind limbs. The bones were cut at both ends, and prewarmed PBS was flushed through the medullary cavity using a syringe fitted with a 27G needle, to extract the bone marrow cells. The resulting cell suspension was filtered through a 100 µm cell strainer and centrifuged at 2500 rpm for 10 min at room temperature. To obtain free amastigotes, the pelleted cells were resuspended within a 75 mL ventilated flask in amastigote culture medium (15 mM KCl; 136 mM KH_2_PO_4_; 10 mM K_2_HPO_4_.3H_2_O; 0.5 mM MgSO_4_.7 H_2_O; 24 mM NaHCO_3_; 22 mM glucose; 1 mM glutamine, 1x RPMI 1640 vitamin mix (Sigma-Aldrich, Merck, Darmstadt, Germany), 10 mM folic acid, 100 mM adenosine, 1x RPMI amino acid mix (Sigma-Aldrich, Merck, Darmstadt, Germany), 5 mg/mL hemin, antibiotic mixture, 25 mM MES, 10% FBS (Gibco, ThermoFisher Scientific, Waltham, MA, USA) and incubated at 37 °C in a humidified atmosphere containing 5% CO_2_ [46]. 

### 4.5. Culture of Ex Vivo Splenic Explants Infected with L. donovani IRFP

The isolation of intramacrophagic *L. donovani* IRPF amastigotes was performed as previously described [47]. Briefly, 1.5 × 10^9^ infectious metacyclic promastigotes were injected intraperitoneally into Balb/c mice. Once infection was established (8–12 weeks post-inoculation), animals were humanely euthanized, and their spleens were aseptically removed, cut into small pieces, and digested with collagenase and biliverdin under sterile conditions. The cell pellet was resuspended in RPMI medium (Gibco) enriched with 20% FBS, 1 mM sodium pyruvate, 24 mM NaHCO_3_, 2 mM L-glutamine, 1x RPMI vitamins, 25 mM HEPES, and antibiotic mixture.

### 4.6. In Vitro Cytotoxicity and Tolerance Assessment

The efficacy of the selected compounds was assessed by measuring near-infrared fluorescence (700 nm) using an Odyssey Infrared Imaging System (Li-Cor, Lincoln, NE, USA) following the previously described protocols [47].

To evaluate the safety of the selected compounds, two cell lines were employed: RAW 264.7 (a mouse macrophage-derived cell line) and HepG2 (a human hepatoma cell line). Furthermore, to determine the intestinal tolerance relevant to potential oral administration, mouse intestinal organoids were prepared.

RAW 264.7 and HepG2 were routinely cultured under standard conditions. RAW 264.7 cells were maintained in RPMI medium (Gibco) supplemented with, 25 mM HEPES, 24 mM NaHCO_3_, 2 mM L-glutamine, mixture of antibiotics and 10% FBS. HepG2 cells were cultured in DMEM/F-12 (Gibco) and supplemented 10% of FBS. For cytotoxicity assays, 10,000 cells per well were seeded into 96-well plates and incubated overnight at 37 °C with 5% of CO_2_. Subsequently, 2-fold serial dilutions of the compounds were added and incubated for 72 h. Cell viability using the alamarBlue™ assay according to the manufacturer’s instructions, and fluorescence was measured with a Varioskan™ LUX microplate reader (ThermoFisher Scientific, Waltham, MA, USA). Non-linear regression analyses were performed using SigmaPlot^TM^ software (version 10.0) to calculate CC_50_. Data were obtained from at least three independent experiments performed in duplicate.

Mouse intestinal organoids were generated following protocols standardized previously [48]. Briefly, to obtain adult intestine stem cells, C57BL/6 mice were humanely euthanized. A section of the duodenum was excised, rinsed in ice-cold PBS, and cut into small pieces for gentle dissociation using Gentle Cell Dissociation Reagent. Tissue fragments enriched in duodenal crypts and little tissue debris were microscopically selected and resuspended in a 1:1 mixture of Geltrex™ and IntestiCult™ Mouse Medium (Gibco, ThermoFisher Scientific, Waltham, MA, USA /Stemcell Technologies^TM^, Vancouver, BC, Canada). Aliquots of 50 µL of this suspension were dispensed into the center of each well of a prewarmed 24-well plate to form domes containing organoids, which were then incubated to allow matrix polymerization. Then, 500 mL of IntestiCult™ Mouse Medium was added to each well. The plate was incubated at 37 °C in a humidified atmosphere containing 5% CO_2_, and the culture medium was refreshed every 48 h. During this period, organoids continued to proliferate, and passaging was performed at a 1:4 ratio every 7–10 days.

To determine the in vitro tolerance to oral administration of the active compounds, a 384-well assay was adapted from the protocol described by Du and co-workers [49]. Briefly, following medium removal, organoids were collected from the extracellular matrix using the same procedure as for passaging. The resulting pellet was resuspended in 3.2 mL of a 1:1 mixture of Geltrex™ LDEV-Free Reduced Growth Factor Basement Membrane Matrix (without phenol red, Gibco) and IntestiCult™ Mouse Medium. Using a chilled pipette dispenser, 8 µL of the organoid suspension was dispensed into pre-cooled 384-well plates, which were then gently agitated to evenly distribute the matrix. The plates were incubated at 37 °C for 10 min and then, 32 µL of IntestiCult™ Mouse Medium was added to each well. To prevent edge effects, 100 µL of sterile water was added to the perimeter wells. After 3 days of culture, 10 µL per well of the test compounds dissolved in IntestiCult™ Mouse Medium were added. Positive (0.03% H_2_O_2_) and negative (0.2% DMSO) controls were included in each assay. After 72 h of incubation, 5 µL of alamarBlue™ HS were added to each well, and fluorescence was measured after 24 h using a Varioskan™ LUX microplate reader. All assays were conducted in triplicate and repeated in at least three independent experiments.

### 4.7. Chemo-Informatics

#### 4.7.1. Chemical Clustering

DataWarrior version V06.04.02—an open-source cheminformatics software tool developed by Sander and coworkers and www.openmolecules.org (accessed on 31 March 2025) [18]—was used to group compounds based on chemical structure similarity. The SMILES of the 72 active compounds were compiled into a .csv file and used to calculate the SkelSpheres molecular descriptors for each molecule in the dataset. Clustering was then performed using the default parameters. The SkelSpheres descriptor is particularly suitable for fine-grained chemical graph similarity analysis, as it accounts for both aromatic and stereochemical features.

#### 4.7.2. Murcko Scaffold Enrichment

The Murcko Scaffold, which represents the core ring systems and the direct connections among them within a molecule, was extracted from the complete list of alkaloids analyzed by the DataWarrior software [23]. To identify enriched Murcko frameworks in the active compound dataset, a two-step filtering approach was applied. First, scaffolds with a frequency greater than 1 were selected. For the enrichment analysis, only those scaffolds containing at least one active compound were retained. The Enrichment Factor (EF) was then calculated to quantify the relative abundance of active compounds within each retained Murcko scaffold. EF was defined as the ratio between the proportion of active compounds within a given scaffold and the overall proportion of active compounds in the dataset [50,51].

#### 4.7.3. Predictive Druglikeness and ADMET

ADMET and druglikeness properties were evaluated using three open-source cheminformatic tools: DataWarrior [18], SwissADME [52] and admetSAR3.0 [53]. In DataWarrior, physicochemical and toxicity-related properties were calculated using the SMILES entered through chemical structure menu. The selected druglikeness parameters included the number of hydrogen bond acceptors (nHA), number of hydrogen bond donors (nHD), number of rotatable bonds (nRot), topological polar surface area (TPSA), lipophilicity (cLogP); water solubility (cLogS), molecular weight (Mw) and overall druglikeness score. The evaluated toxicity parameters comprised mutagenic, tumorigenic, reproductive effects, irritation and PAINS (Pan-Assay Interference Compounds) patterns. In the SwissADME, the SMILES of our six compounds were introduced to obtain additional data on physicochemical properties, lipophilicity, water solubility, pharmacokinetic behaviour, druglikeness and medicinal chemistry features. Finally, the same SMILES strings were uploaded to the admetSAR3.0 platform to predict ADMET profiles.

## 5. Conclusions

Screening of the commercial alkaloid collection HY L071 identified a group of 16 compounds with antileishmanial activity and well-defined chemical scaffolds. In silico prediction for the six most promising compounds—those with the highest efficacy, safety, and intestinal tolerability—revealed that all were derived from isoquinoline—one benzo[c]phenanthridine and five protoberberines. These compounds were predicted to be suitable for oral administration and, with the exception of angoline, did not present significant toxicological problems for further development.

## Figures and Tables

**Table 1:** Efficacy and safety of the 16 compounds that reduce the viability of *L. donovani* IRFP by ≥ 70% at 10 µM or 1µM in at least one experimental replicate.

Product Name	IC_50_ Axenic Amastigotes (µM)	EC_50_ Intramacrophagic Amastigotes (µM)	CC_50_ RAW 264.7 (µM)	SI_1_	CC_50_ HepG2 (µM)	SI_2_
Dihydrochelerythrine	0.06 ± 0.005	10.49 ± 0.75	>50	>4.8	>50	>4.8
Ethoxysanguinarine	0.21 ± 0.03	0.84 ± 0.03	1.62 ± 0.11	1.9	1.44 ± 0.03	1.7
Angoline	0.12 ± 0.01	1.15 ± 0.04	5.28 ± 0.28	4.6	5.15 ± 0.21	4.5
Dihydrosanguinarine	0.19 ± 0.01	8.55 ± 1.33	46.50 ± 10.07	5.4	>40	>4.7
Chelerythrine	0.08 ± 0.01	1.61 ± 0.05	5.51 ± 0.16	3.4	5.50 ± 0.64	3.4
Sanguinarine	0.19 ± 0.01	0.85 ± 0.03	1.84 ± 0.06	2.2	2.08 ± 0.06	2.4
Nitidine	0.27 ± 0.01	0.65 ± 0.03	2.01 ± 0.16	3.1	0.49 ± 0.02	0.7
Berberine	0.57 ± 0.08	1.76 ± 0.06	>20	>11.4	>20	>11.4
13-Methylberberine	0.50 ± 0.04	0.75 ± 0.02	>10	>13.3	28.27 ± 9.18	37.7
Dehydrocorydaline	2.81 ± 0.49	2.68 ± 0.11	>10	>3.7	>10	>3.7
Coptisine	2.85 ± 0.25	3.39 ± 0.23	>10	>2.9	>10	>2.9
Pseudocoptisine	0.81 ± 0.12	1.43 ± 0.07	>10	>7.0	>10	>7.0
Neotuberostemonine	1.27 ± 0.06	>25	39.14 ± 2.2	>1.6	>50	>2.0
Tuberostemonine	2.06 ± 0.11	>50	50.04 ± 5.51	>1.0	>40	>0.8
Dehydronuciferine	1.23 ± 0.03	>50	18.59 ± 1.11	>0.37	25.54 ± 5.43	>0.51
Meisoindigo	1.53 ± 0.21	9.30 ± 0.52	14.91 ± 0.90	1.6	20.44 ± 1.40	2.2

SI_1_ calculated between RAW 264.7 cells and intramacophagic amastigotes. SI_2_ calculated between HepG2 cells and intramacrophagic amastigotes.

**Table 2:** Predictive druglikeness and ADMET of the six compounds meeting the lead criteria of EC_50_ < 3 µM and SI ≥ 4 identified in the alkaloid library (MedChemExpress, Sollentuna, Sweden. Ref.: HY−L071).

	Berberine		13-Methylberberine		Angoline		Dehydrocorydaline		Pseudocoptisine		Coptisine	
	Swiss ADME	Admet SAR3.0	Swiss ADME	Admet SAR3.0	Swiss ADME	Admet SAR3.0	Swiss ADME	Admet SAR3.0	Swiss ADME	Admet SAR3.0	Swiss ADME	Admet SAR3.0
**Druglikeness rules**	**Result**	**Prob.**	**Result**	**Prob.**	**Result**	**Prob.**	**Result**	**Prob.**	**Result**	**Prob.**	**Result**	**Prob.**
Lipinski rule	NV	1	NV	1	NV	1	NV	1	NV	1	NV	1
**Absorption**	**Result**	**Prob.**	**Result**	**Prob.**	**Result**	**Prob.**	**Result**	**Prob.**	**Result**	**Prob.**	**Result**	**Prob.**
GI/HIA	High	0.96	High	0.98	High	0.98	High	0.98	High	0.99	High	0.98
BBB	No	0.96	Yes	0.98	Yes	0.96	Yes	0.94	No	0.98	No	0.98
**Metabolism**	**Result**	**Prob.**	**Result**	**Prob.**	**Result**	**Prob.**	**Result**	**Prob.**	**Result**	**Prob.**	**Result**	**Prob.**
CYP450 1A2 inhibitor	Yes	0.59	No	0.86	Yes	0.92	No	0.55	Yes	0.91	Yes	0.89
CYP450 2C9 inhibitor	No	0.28	No	0.50	Yes	0.60	No	0.17	No	0.64	No	0.63
CYP450 2D6 inhibitor	No	0.29	Yes	0.27	Yes	0.17	Yes	0.14	No	0.38	No	0.36
CYP450 2C19 inhibitor	No	0.56	No	0.77	Yes	0.85	No	0.44	No	0.87	No	0.88
CYP450 3A4 inhibitor	No	0.22	No	0.23	Yes	0.69	Yes	0.11	No	0.34	No	0.32
	Data Warrior	Admet SAR3.0	Data Warrior	Admet SAR3.0	Data Warrior	Admet SAR3.0	Data Warrior	Admet SAR3.0	Data Warrior	Admet SAR3.0	Data Warrior	Admet SAR3.0
**Toxicity**	**Result**	**Prob.**	**Result**	**Prob.**	**Result**	**Prob.**	**Result**	**Prob.**	**Result**	**Prob.**	**Result**	**Prob.**
AMES test	None	0.68	None	0.76	High	0.92	None	0.67	None	0.81	None	0.86
Carcinogenicity	None	0.52	None	0.56	High	0.77	None	0.57	None	0.63	None	0.58

NV = No Violation; GI/HIA = Human Gastrointestinal absorption; BBB = Blood–brain barrier; Prob. = probability. Toxicity: Rodent carcinogenicity in admetSAR3.0 and just carcinogenicity in DataWarrior.

## Data Availability

The original contributions presented in this study are included in the article/Supplementary Material. Further inquiries can be directed to the corresponding author.

## Supplementary Materials

The following supporting information can be downloaded at: https://www.mdpi.com/article/10.3390/molecules30214210/s1, Figure S1: Z’-factor; Figure S2: 2D similarity chart; Figure S3: Dose–response curves of the remaining hit compounds; Figure S4: Dose–response curves of the lead compounds in organoids; Table S1: List of the 72 hit compounds; Table S2: Total Murcko scaffolds. Table S3: Other druglikeness rules; Table S4: List of 449 alkaloids compounds tested.

## Abbreviations

The following abbreviations are used in this manuscript:

NTD	Neglected Tropical Diseases
VL	Visceral Leishmaniasis
HTS	High-Throughput Screening
IRPF	Infrared Fluorescent Protein
DNDi	Drugs for Neglected Diseases Initiative
FBS	Fetal Bovine Serum
ADMET	Absorption, Distribution, Metabolism, Excretion and Toxicity
SMILES	Simplified Molecular Input Line Entry System
PAINS	Pan-Assay Interference Compounds
