# Supplementary material for: Discovery of Potential Antileishmanial Compounds Through Phenotypic Screening of an Alkaloid Library
Source: Molecules. 2025 Oct 28;30(21):4210. doi: 10.3390/molecules30214210 (PMC12608155; doi:10.3390/molecules30214210)
Supplement: Supplementary file 1 [file molecules-30-04210-s001.zip › molecules-3923261-supplementary.pdf]

SUPPLEMENTARY MATERIALS

Table S1. List of the 72 alkaloids that show antileishmanial activity in axenic amastigotes of *L. donovani* IRFP at 10 μM or 1μM

		Growth Inhibition (%) at 72h			
**ProductName**	**CAS Number**	**10 μM_Exp1**	**10 μM_Exp2**	**1 μM_Exp1**	**1 μM_Exp2**
Dehydronuciferine	7630-74-2	100.28	100.10	86.68	77.77
Harringtonine	26833-85-2	92.52	92.20	-25.30	3.29
Halofuginone (hydrobromide)	64924-67-0	100.16	98.51	99.34	83.65
Dehydrocorydaline (hydroxyl)		92.43	77.86	10.19	-0.13
Staurosporine	62996-74-1	100.05	99.79	96.35	98.74
Tryptanthrin	13220-57-0	84.08	74.98	13.06	23.36
Nordihydrocapsaicin	28789-35-7	90.15	70.24	1.91	-21.65
Tabersonine	4429-63-4	98.00	61.50	-26.17	-7.79
Indole-3-butyric acid	133-32-4	70.17	83.99	-6.27	14.84
Cyclanoline (chloride)	17472-50-3	77.56	17.25	-48.98	13.86
Dihydroevocarpine	15266-35-0	97.61	83.00	-21.58	19.18
Meisoindigo	97207-47-1	100.05	100.02	99.95	100.00
Camptothecin	7689-03-4	90.95	95.89	66.41	89.30
Anisodamine (hydrobromide)	55449-49-5	80.63	11.58	-15.73	4.55
(-)-Alkannin	517-88-4	99.90	99.93	100.44	100.08
Berberine (chloride)	633-65-8	99.29	97.39	16.68	6.56
9-Aminocamptothecin	91421-43-1	74.70	89.10	-3.99	61.95
Cyclopiazonic acid	18172-33-3	100.43	100.09	20.82	19.94
Dehydrocorydaline (chloride)	10605-03-5	95.47	88.17	13.00	31.86
Dihydrocapsaicin	19408-84-5	91.98	71.10	-4.08	35.31
Lobelanine	579-21-5	75.56	20.66	-73.70	10.47
Piperlongumine	20069-09-4	100.21	100.05	99.95	98.63
Febrifugine	24159-07-7	99.75	98.19	15.43	-25.30
Dihydrochelerythrine	6880-91-7	100.32	100.11	100.31	97.914
Berberine (sulfate)	633-66-9	70.74	94.59	19.57	-24.39
Tomatidine	77-59-8	66.93	75.07	11.24	18.61
Ellipticine (hydrochloride)	5081-48-1	98.68	97.67	36.30	-10.32
13-Methylberberine (chloride)	54260-72-9	86.05	98.60	58.33	69.84
Robustine	2255-50-7	90.57	33.27	48.34	1.86
Vinblastine (sulfate)	143-67-9	99.34	78.77	-20.42	-25.23
Ethoxysanguinarine	28342-31-6	100.41	100.07	100.74	99.44
Solamargine	20311-51-7	93.59	94.33	62.77	11.21
Piperine	94-62-2	-7.31	75.21	38.33	17.53
2,3-Bis(3-indolylmethyl) indole	138250-72-3	100.59	100.36	-5.15	13.55

Angoline	21080-31-9	100.85	100.07	83.55	85.33
Pyridoxine (hydrochloride)	58-56-0	79.17	65.78	6.77	-86.02
Chelerythrine	3895-92-9	100.46	100.71	97.15	95.70
Halofuginone	55837-20-2	97.69	98.17	93.66	90.93
N-Benzyllinoleamide	18286-71-0	99.77	99.86	23.06	-24.6
Cephaeline (dihydrochloride)	5853-29-2	96.96	92.99	-15.97	-74.54
Evodiamine	518-17-2	94.67	96.80	-42.64	25.77
Dihydrosanguinarine	3606-45-9	99.83	99.70	99.27	97.59
Coptisine (chloride)	6020-18-4	84.96	96.73	29.53	16.08
Dehydrocorydaline	30045-16-0	81.68	88.30	19.06	-42.59
Narciclasine	29477-83-6	84.56	93.45	-12.26	-86.55
Corydaline	518-69-4	79.13	87.39	-15.75	2.30
Homoharringtonine	26833-87-4	89.91	91.50	4.68	-119.25
N-Benzyllinolenamide	883715-18-2	99.95	100.85	-37.93	-43.83
Maleimide	541-59-3	100.79	100.81	100.90	101.28
Phenazine-1-carboxylic acid	2538-68-3	90.84	87.83	-12.19	-87.03
Febrifugine (dihydrochloride)	32434-42-7	97.63	97.12	74.39	25.76
9-Methoxycamptothecin	39026-92-1	96.02	94.80	64.49	73.97
Tuberostemonine	6879-01-2	101.58	101.30	63.61	53.024
Berberine (chloride hydrate)	68030-18-2	98.55	99.87	29.91	24.54
3,3'-Diindolylmethane	1968-05-4	100.37	95.74	-59.46	-44.58
3-Indoleacetic acid	87-51-4	101.77	101.49	-25.43	-9.83
Rubitecan	91421-42-0	94.36	89.43	69.82	36.86
Nonivamide	2444-46-4	101.01	93.27	10.88	-30.69
Paxilline	57186-25-1	98.63	99.06	6.12	19.65
12-Ethyl-9-hydroxycamptothecin	119577-28-5	70.30	62.49	22.17	56.54
Dehydrocorydaline (nitrate)	13005-09-9	82.98	66.56	27.42	-14.19
(±)-Evodiamine	518-18-3	100.52	97.39	64.72	40.81
7-Ethylcamptothecin	78287-27-1	96.57	91.90	70.44	77.88
10-Methoxycamptothecin	19685-10-0	97.42	93.76	73.40	85.61
Evocarpine	15266-38-3	100.35	98.71	-18.62	-35.10
Neotuberostemonine	143120-46-1	100.59	101.12	37.98	69.73
Iminodiacetic acid	142-73-4	-16.89	79.93	-16.61	-73.05
Sanguinarine (chloride)	5578-73-4	100.39	100.55	99.70	99.54
Pseudocoptisine (chloride)	30044-78-1	97.95	99.04	65.54	88.42
Coptisine (Sulfate)	1198398-71-8	71.50	83.90	-9.12	-56.10
Nitidine (chloride)	13063-04-2	100.40	99.74	45.01	69.56
Demethyleneberberine (chloride)	16705-03-6	43.20030465	71.18	-4.24	8.25

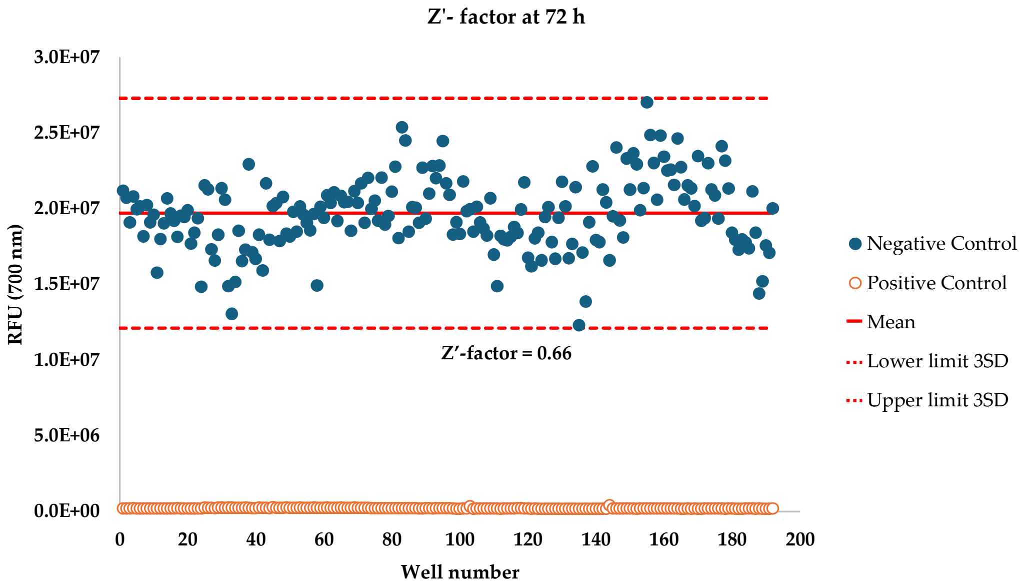

Figure S1. Z′-Factor value of plates containing the tested alkaloids. RFU (Relative Fluorescence Units); SD (Standard Deviation).

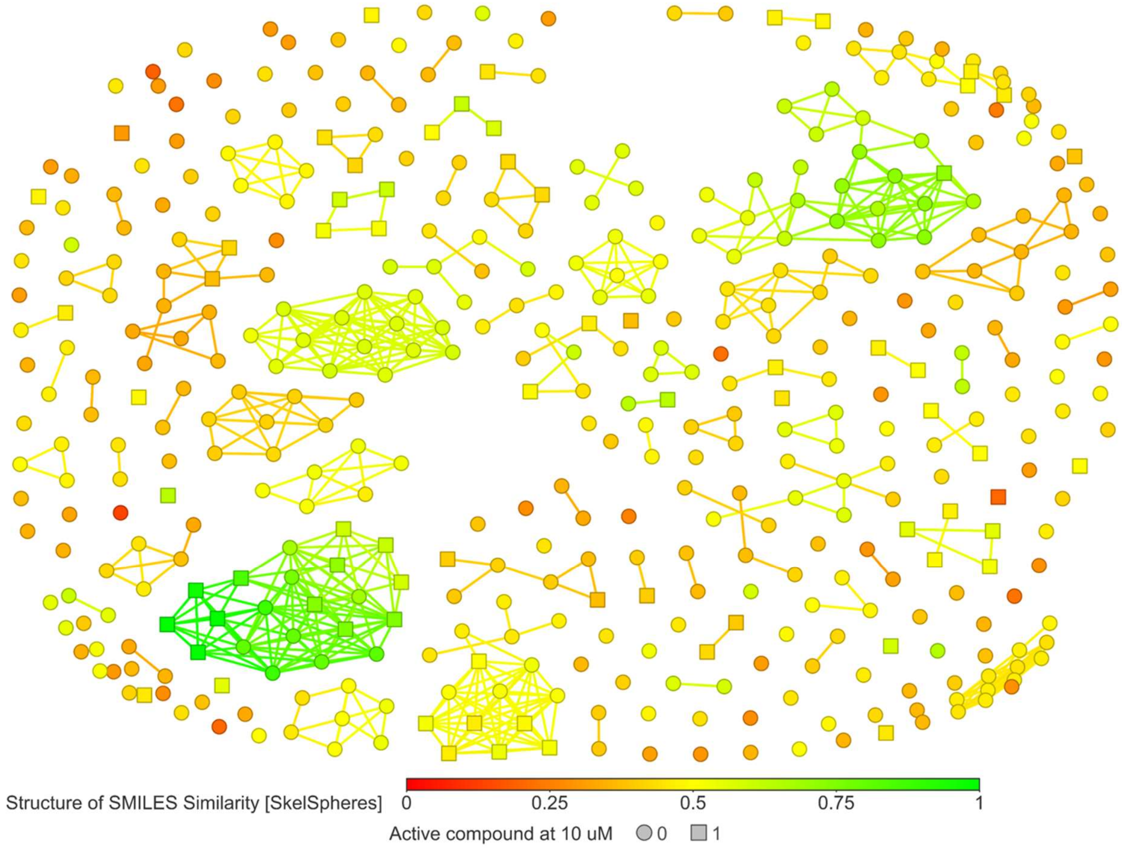

Figure S2. 2D similarity chart of 449 alkaloids of the HY-L071 library (MedChemExpress) used in the phenotypic screening. Markers are dynamically colored based on structural similarity to the selected reference compound dehydrocorydaline. Similar neighbors are connected by lines.

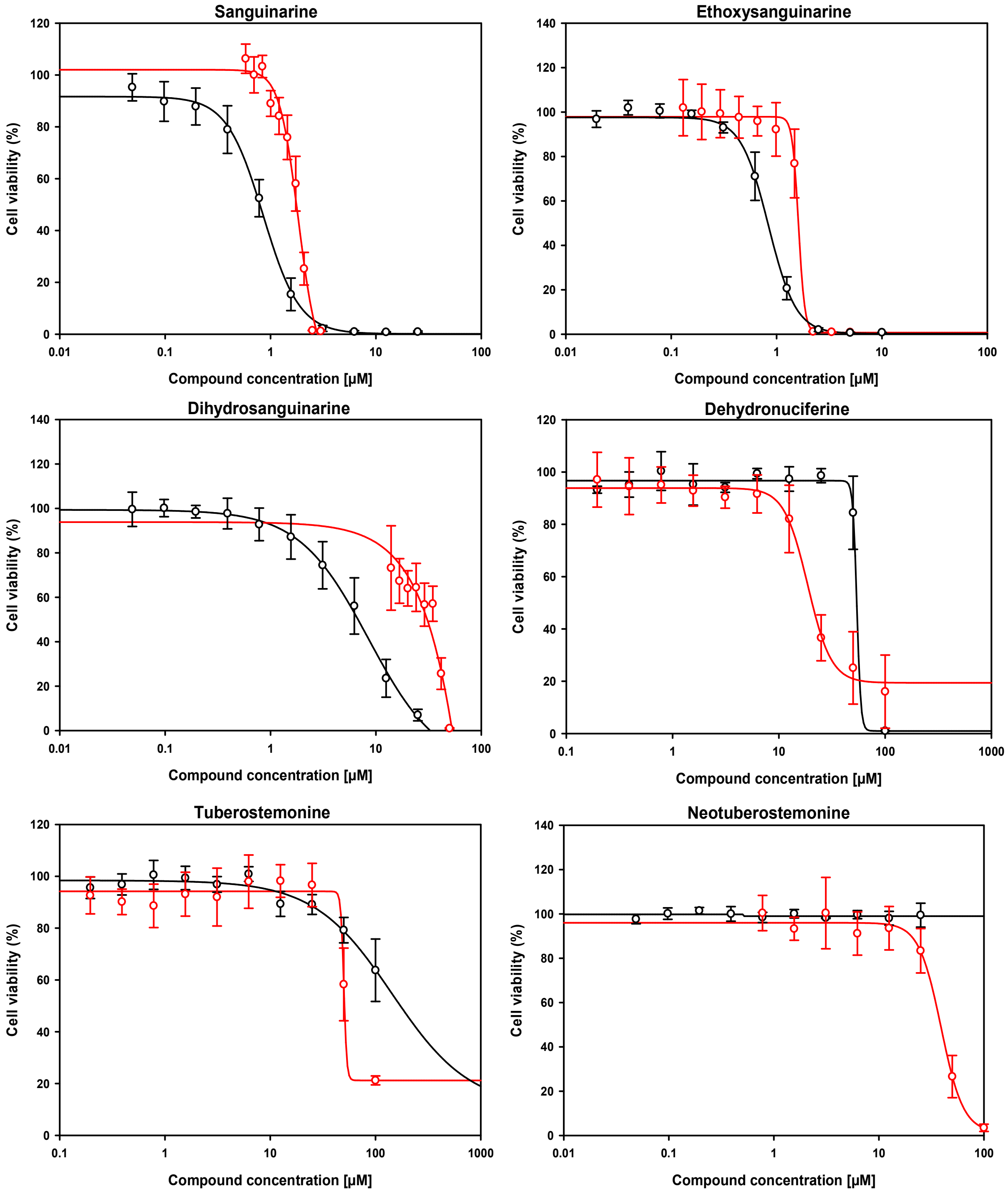
Sanguinarine
Ethoxysanguinarine
Dihydrosanguinarine
Dehydronuciferine
Tuberostemonine
Neotuberostemonine
Cell viability (%)
Compound concentration [µM]
0
20
40
60
80
100
120
140
0.01
0.1
1
10
100
1000

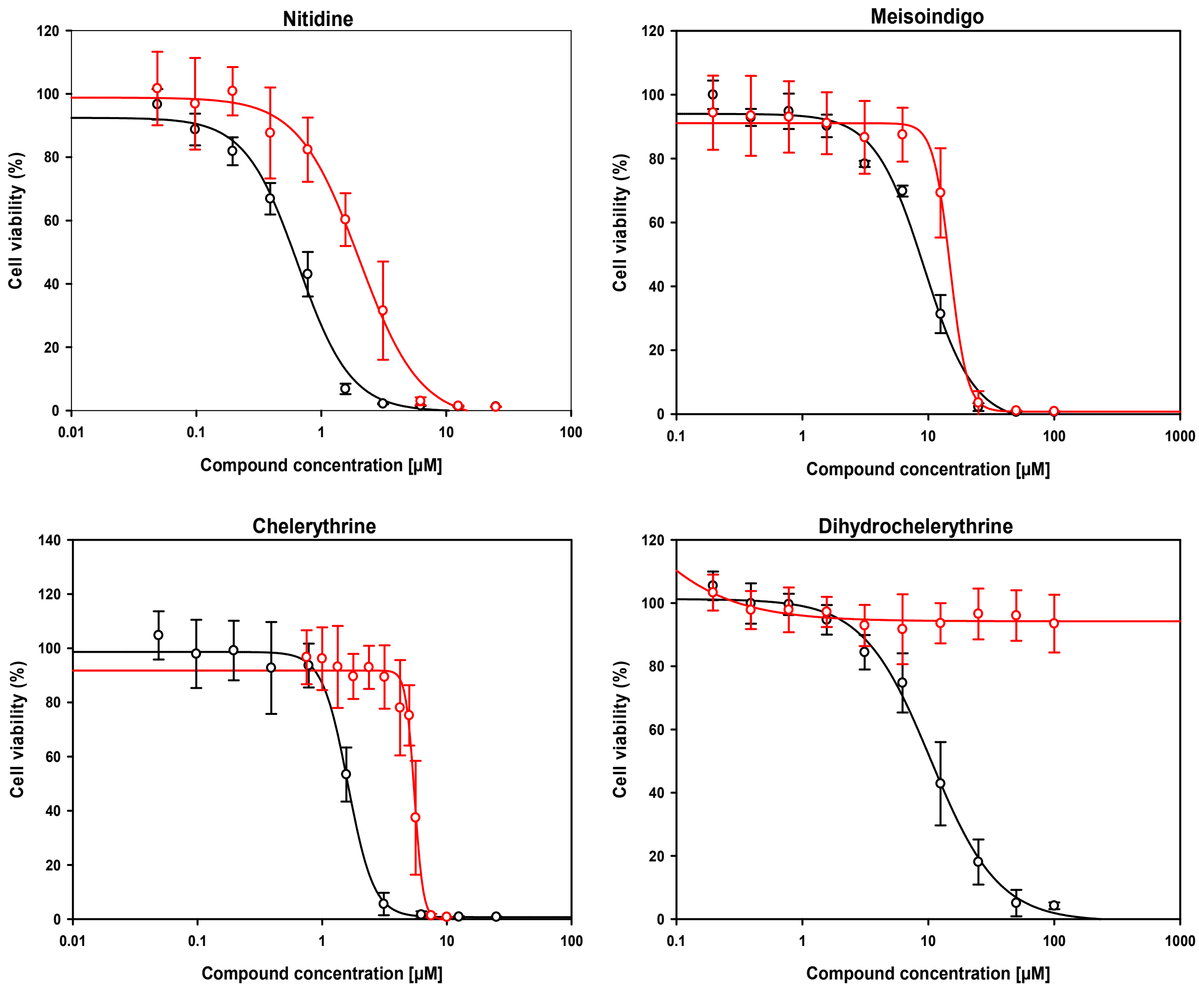

Figure S3. Dose-response curves of the remaining hit compounds identified as potential hits from the Alkaloids Compound Library (MedChemExpress HY-L071) on intramacrophagic amastigotes in primary cultures of infected mouse splenocytes and the cytotoxic effect in RAW 264.7 mouse macrophages are shown in black and red respectively. EC_{50} and CC_{50} values, present in Table 1 of the manuscript, were calculated using the SigmaPlot™ statistical software version 10.0. Each point represents the mean ± SD of at least three different experiments performed in triplicate.

Table S2. Total Murcko scaffolds found and representative alkaloids that have anti-leishmanial activity, generated using the criteria described in the Materials and Methods Section.

NO.	MURCKO SCAFFOLD	TC	AC	IC	AF	EF	ACTIVE COMPOUNDS NAMES
1	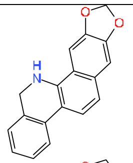	2	2	0	1	6.25	Dihydrochelerythrine; Angoline
2	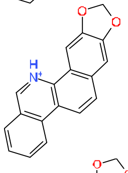	2	2	0	1	6.25	Chelerythrine ; (chloride); Nitidine (chloride)
3	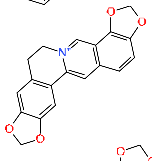	2	2	0	1	6.25	Coptisine (chloride); Coptisine (Sulfate)
4	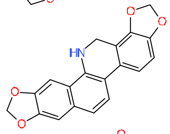	2	2	0	1	6.25	Dihydrosanguinarine; Ethoxysanguinarine
5	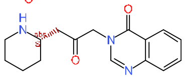	2	2	0	1	6.25	Febrifugine; Febrifugine (dihydrochloride)
6	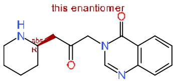	2	2	0	1	6.25	Halofuginone (hydrobromide); Halofuginone
7	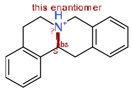	2	1	1	0.50	3.13	Cyclanoline (chloride)
8	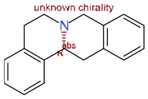	2	1	1	0.50	3.13	Corydaline
9	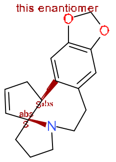	3	2	1	0.67	4.19	Harringtonine; Homoharringtonine
10	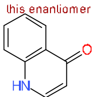	4	2	2	0.50	3.13	Dihydroevocarpine; Evocarpine
11	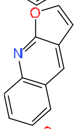	4	1	3	0.25	1.56	Robustine
12	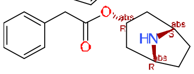	4	1	3	0.25	1.56	Anisodamine (hydrobromide)
13	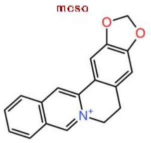	5	4	1	0.80	5	Berberine (chloride hydrate); Berberine (chloride); Berberine (sulfate); 13-Methylberberine (chloride)

14	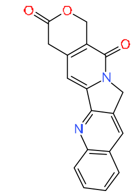	11	7	4	0.64	4	9-Aminocamptothecin; 12-Ethyl-9-hydroxycamptothecin; Camptothecin; Rubitecan; 7-Ethylcamptothecin; 9-Methoxycamptothecin; 10-Methoxycamptothecin
15	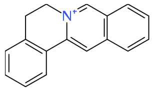	11	5	6	0.45	2.81	Demethyleneberberine (chloride); Dehydrocorydaline; Dehydrocorydaline (chloride); Dehydrocorydaline (hydroxyl); Dehydrocorydaline (nitrate)
16	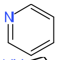	12	1	11	0.08	0.5	Pyridoxine (hydrochloride)
17	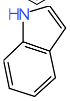	14	2	12	0.14	0.87	3-Indoleacetic acid; Indole-3-butyric acid
18	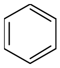	20	5	15	0.25	1.56	Nordihydrocapsaicin; Dihydrocapsaicin; N-Benzyllinolenamide; N-Benzyllinoleamide; Nonivamide

TC: Total Compound; AC: Active Compound; IC: Inactive Compound; AF: Active Fraction; EF: Enrichment Factor.

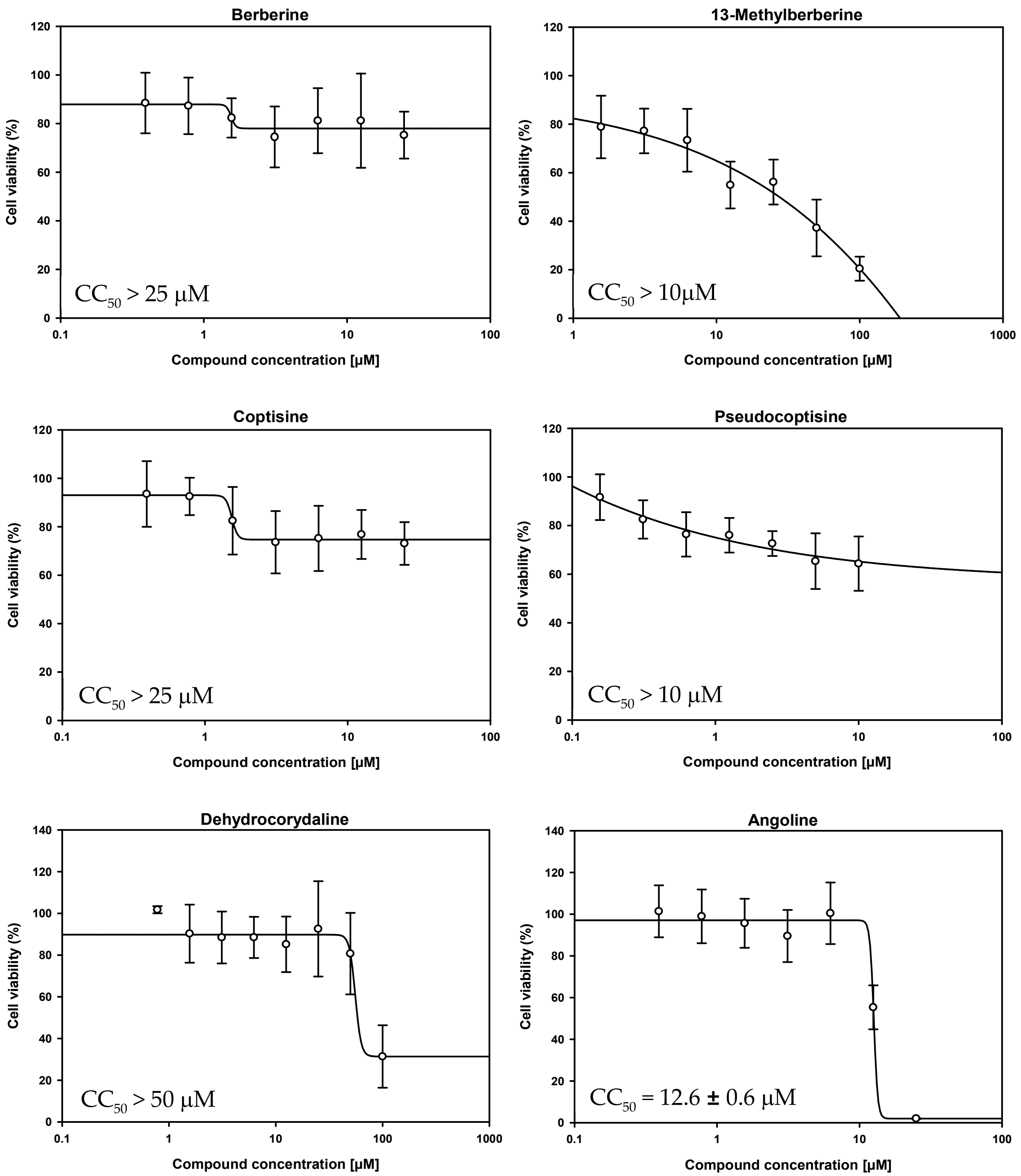

Figure S4. Dose-response curves of the tolerance of mouse intestinal organoids to the the six lead compounds identified in our study.

Table S3. Druglikeness parameters with Ghose, Veber, Egan, Muegge, Pfizer and GSK rules obtained from SwissADME and admetSAR3.0 tools.

	**Berberine**		**13-Methylberberine**		**Angoline**		**Dehydrocorydaline**		**Pseudocoptisine**		**Coptisine**	
	Swiss ADME	Admet SAR3.0	Swiss ADME	Admet SAR3.0	Swiss ADME	Admet SAR3.0	Swiss ADME	Admet SAR3.0	Swiss ADME	Admet SAR3.0	Swiss ADME	Admet SAR3.0
Druglikeness rules	Result	Prob.	Result	Prob.	Result	Prob.	Result	Prob.	Result	Prob.	Result	Prob.
Ghose rule	NV	-	NV	-	NV	-	NV	-	NV	-	NV	-
Veber rule	NV	-	NV	-	NV	-	NV	-	NV	-	NV	-
Egan rule	NV	-	NV	-	NV	-	NV	-	NV	-	NV	-
Muegge rule	NV	-	NV	-	NV	-	NV	-	NV	-	NV	-
Pfizer rule	-	NA	-	NA	-	NA	-	NA	-	A	-	A
GSK rule	-	NA	-	NA	-	NA	-	A	-	A	-	A

NV= No Violation; NA= Not Accepted; A= Accepted

Table S4. List of the 449 compounds tested from HY-L071 alkaloids library (MedChemExpress).

Catalog Number	ProductName	CAS Number
HY-B1751	Quinidine (15% dihydroquinidine)	56-54-2
HY-B1167	Ajmaline	4360-12-7
HY-W002168	1,3-Dimethylpyrazole	694-48-4
HY-W001132	Indole	120-72-9
HY-N0930B	Galegine (hydrochloride)	2368870-39-5
HY-N0478	Neoline	466-26-2
HY-N0096	Rotundine	483-14-7
HY-N0471A	L-Hyoscyamine (sulfate)	620-61-1
HY-113262	8-Hydroxyguanosine	3868-31-3
HY-N0288	Lycorine	476-28-8
HY-Y0061	Oxindole	59-48-3
HY-113239	Hydroxycotinine	34834-67-8
HY-N1372	(R)-Fangchinoline	33889-68-8
HY-W010195	2,6-Dimethylquinoline	877-43-0
HY-N2322	Khasianine	32449-98-2
HY-B1205	Atropine	51-55-8
HY-N2352A	Laurolitsine (hydrochloride)	
HY-15122	Sinomenine	115-53-7
HY-N1974	Fuziline	80665-72-1
HY-N6691	Veratridine	71-62-5
HY-N1402	Oxysophoridine	54809-74-4
HY-N0924A	(±)-Stylopine (hydrochloride)	96087-21-7
HY-N0071	Crotonoside	1818-71-9
HY-N4261	Dehydronuciferine	7630-74-2
HY-N7263	Galanthamine N-Oxide	134332-50-6
HY-121936A	Yohimbic acid (hydrate)	207801-27-2
HY-N0862	Harringtonine	26833-85-2
HY-Y0265	Isatin	91-56-5
HY-N1882	4,5-Dimethoxycanthin-6-one	18110-87-7
HY-N0450	Sinapine (thiocyanate)	7431-77-8
HY-N6857	Armepavine	524-20-9
HY-N0714A	Berbamine (dihydrochloride)	6078-17-7
HY-B0979	Lobeline (hydrochloride)	134-63-4
HY-B0997	Hydroquinidine	1435-55-8
HY-N0740	Jatrorrhizine (chloride)	6681-15-8
HY-N1584A	Halofuginone (hydrobromide)	64924-67-0
HY-100971	Spiramide	510-74-7
HY-113081	1-Methyladenosine	15763-06-1
HY-N6638	Retrorsine	480-54-6
HY-N2410	N-trans-Feruloyltyramine	66648-43-9
HY-17024	Cyclopamine	4449-51-8
HY-N0413	Hupehenine	98243-57-3
HY-N4068	Glucoraphanin	21414-41-5
HY-W018800	4(3H)-Quinazolinone	491-36-1
HY-N2043	Huperzine B	103548-82-9
HY-N2625A	Harmalol (hydrochloride)	6028-07-5
HY-B0927	Hydrastine	118-08-1
HY-17387	(-)-Huperzine A	102518-79-6
HY-N0132A	Synephrine (hydrochloride)	5985-28-4
HY-A0009	Galanthamine (hydrobromide)	1953-04-4
HY-N0275	(±)-10-Hydroxycamptothecin	64439-81-2
HY-N0674B	Dehydrocorydaline (hydroxyl)	

HY-N0329	Deltaline	6836-11-9
HY-N0443	N-Methylcytisine	486-86-2
HY-N9404	6-Benzoylheteratisine	99759-48-5
HY-N0826	Corynoline	18797-79-0
HY-W012185	(-)-Sparteine	90-39-1
HY-N7271	Solanidine	80-78-4
HY-W042156	Aegeline	456-12-2
HY-N6973	Boldine	476-70-0
HY-129583	Lepidiline A	596093-98-0
HY-16562A	Irinotecan (hydrochloride)	100286-90-6
HY-15141	Staurosporine	62996-74-1
HY-N0849	Dictamine	484-29-7
HY-N6607	Tryptanthrin	13220-57-0
HY-135005	Biliverdin (hydrochloride)	856699-18-8
HY-124124	N-Methylnicotinamide	114-33-0
HY-N2064	Racanisodamine	17659-49-3
HY-W091541	4-Hydroxy-1H-indole-3-carbaldehyde	81779-27-3
HY-N0441	Neferine	2292-16-2
HY-113440	5-Methoxytryptophol	712-09-4
HY-W016887	H-Gly-Pro-OH	704-15-4
HY-N0220	Dauricine	524-17-4
HY-B1304A	(+)-Sparteine (sulfate pentahydrate)	
HY-N0449	Nordihydrocapsaicin	28789-35-7
HY-N0584	Anisodamine	55869-99-3
HY-N2030	Perakine	4382-56-3
HY-N1431	Tabersonine	4429-63-4
HY-122524	7-Methylguanosine	20244-86-4
HY-12053A	Vinorelbine (ditartrate)	125317-39-7
HY-A0066A	Tolazoline (hydrochloride)	59-97-2
HY-N0132	Synephrine	94-07-5
HY-N1103A	Vasicine (hydrochloride)	7174-27-8
HY-N0186	Indole-3-butyric acid	133-32-4
HY-N0741A	Leonurine (hydrochloride)	24735-18-0
HY-N6825	Hydroxy-α-sanshool	83883-10-7
HY-N0095	(S)-10-Hydroxycamptothecin	19685-09-7
HY-D0143	Quinine	130-95-0
HY-125850	Berberrubine (chloride)	15401-69-1
HY-19332	Kifunensine	109944-15-2
HY-N10127	Lepidiline C	2750933-59-4
HY-N0267	Hypaconitine	6900-87-4
HY-113412A	3-Methylhistamine (dihydrochloride)	36475-47-5
HY-N0927	(-)-Isocorypalmine	483-34-1
HY-N4307	Laetanine	72361-67-2
HY-I0736	Isonicotinic acid	55-22-1
HY-N0333	Yunaconitine	70578-24-4
HY-14248	Letrozole	112809-51-5
HY-N2022	Castanospermine	79831-76-8
HY-N7649	Rhombifoline	529-78-2
HY-N0850	Benzoylhypaconine	63238-66-4
HY-N2003	D-Tetrahydropalmatine	3520-14-7
HY-N0654	Corypalmine	27313-86-6
HY-N9452	Capsaicin β-D-glucopyranoside	153409-16-6
HY-N4205	Tetrahydropiperine	23434-88-0
HY-120692	Cyclanoline (chloride)	17472-50-3
HY-122267	Clovamide	53755-02-5

HY-N0737	Harmine (hydrochloride)	343-27-1
HY-W002339	3-Hydroxy-2-methylpyridine	1121-25-1
HY-N2517	Dihydroevocarpine	15266-35-0
HY-N5121	Calycanthine	595-05-1
HY-N0770	Isoliensinine	6817-41-0
HY-76299	Galanthamine	357-70-0
HY-N0103	Sophocarpine	6483-15-4
HY-128699	D-Desthiobiotin	533-48-2
HY-13680	Meisoindigo	97207-47-1
HY-N7596	Isoverticine	23496-43-7
HY-B0573B	Propranolol	525-66-6
HY-B0150	Nicotinamide	98-92-0
HY-16560	Camptothecin	7689-03-4
HY-N7203	N-Caffeoyl O-methyltyramine	189307-47-9
HY-N0049	Nuciferine	475-83-2
HY-N0480A	Reserpine (hydrochloride)	16994-56-2
HY-N0584A	Anisodamine (hydrobromide)	55449-49-5
HY-N1373	Sophoridine	6882-68-4
HY-121118	Coronaridine	467-77-6
HY-N6012	(-)-Alkannin	517-88-4
HY-B0573	Propranolol (hydrochloride)	318-98-9
HY-18258	Berberine (chloride)	633-65-8
HY-N0170	Indole-3-carbinol	700-06-1
HY-100309	9-Aminocamptothecin	91421-43-1
HY-121793	Roemerine	548-08-3
HY-N0214	Peimisine	19773-24-1
HY-116474	Viridicatol	14484-44-7
HY-W040790	2,6-Dimethylpyrazine	108-50-9
HY-B1181	Hydrastinine (hydrochloride)	4884-68-8
HY-N6828	Monocrotaline N-Oxide	35337-98-5
HY-107854	N-Acetyl-5-hydroxytryptamine	1210-83-9
HY-N0219	Bicuculline	485-49-4
HY-65008	N-Demethylricinine	21642-98-8
HY-N7004	Arborine	6873-15-0
HY-N0775	Isocorynoxeine	51014-29-0
HY-N6771	Cyclopiazonic acid	18172-33-3
HY-N0696	Sipeimine	61825-98-7
HY-N0674A	Dehydrocorydaline (chloride)	10605-03-5
HY-19474	Maytansinol	57103-68-1
HY-128553	Antineoplaston A10	91531-30-5
HY-W021267	3-Demethylcolchicine	7336-33-6
HY-N0361	Dihydrocapsaicin	19408-84-5
HY-W052144	(±) Anabasine	13078-04-1
HY-121027	Anagyrine	486-89-5
HY-N2037A	Higenamine (hydrochloride)	11041-94-4
HY-N3035	Tetrahydroepiberberine	38853-67-7
HY-N8505	Lobelanine	579-21-5
HY-N1440	Koumine	1358-76-5
HY-N2329	Piperlongumine	20069-09-4
HY-W008566	Norharmane	244-63-3
HY-N2384	Febrifugine	24159-07-7
HY-N0903	Dihydrochelerythrine	6880-91-7
HY-N5016	Guvacoline (hydrochloride)	6197-39-3
HY-B0809	Theophylline	58-55-9
HY-126562	Piperlonguminine	5950-12-9

HY-B1180	Vinburnine	4880-88-0
HY-N0586	Norisoboldine	23599-69-1
HY-N0716B	Berberine (sulfate)	633-66-9
HY-B0188A	Mianserin (hydrochloride)	21535-47-7
HY-U00082	Tigloidin	495-83-0
HY-B1181A	Hydrastinine	6592-85-4
HY-N2149	Tomatidine	77-59-8
HY-N2392	Kukoamine A	75288-96-9
HY-N0738	Stachydrine hydrochloride	4136-37-2
HY-100807	Quinolinic acid	89-00-9
HY-111914A	Ferroheme	14875-96-8
HY-N2403	Dihydrolycorine	6271-21-2
HY-W007426	N-Methylbenzylamine	103-67-3
HY-N2591	Isocorydine	475-67-2
HY-13295	Vinpocetine	42971-09-5
HY-N0735	Phellodendrine (chloride)	104112-82-5
HY-N2081	Skimmianine	83-95-4
HY-N0117	Indirubin	479-41-4
HY-N0173	Cinchonidine	485-71-2
HY-15753A	Ellipticine (hydrochloride)	5081-48-1
HY-W015818	2-Benzoxazolinone	59-49-4
HY-W001160	5-Hydroxyindole	1953-54-4
HY-131413	O-Desmethyl Galanthamine	60755-80-8
HY-W012956	2-Acetylpyrrole	1072-83-9
HY-N0334A	(+)-Magnoflorine (iodide)	4277-43-4
HY-N2179	Hypaphorine	487-58-1
HY-N0050	Allomatrine	641-39-4
HY-125827	13-Methylberberine (chloride)	54260-72-9
HY-15122A	Sinomenine hydrochloride	6080-33-7
HY-B0394	Atropine (sulfate monohydrate)	5908-99-6
HY-N1343	Robustine	2255-50-7
HY-100385	Brevianamide F	38136-70-8
HY-N7031	(±)-Vasicine	6159-56-4
HY-N2093	Vicine	152-93-2
HY-13780	Vinblastine (sulfate)	143-67-9
HY-W016784	Indole-3-acetamide	879-37-8
HY-N6619A	Lycoramine	21133-52-8
HY-N0103A	Sophocarpine (monohydrate)	145572-44-7
HY-N0252A	Catharanthine (Tartrate)	4168-17-6
HY-W047478	3-Methylcarbazole	4630-20-0
HY-N1955	Protostemotinine	169534-85-4
HY-101392	Harmane	486-84-0
HY-42034	Hydroquinine	522-66-7
HY-N0070	Solasonine	19121-58-5
HY-N2255	Crebanine	25127-29-1
HY-N5009	Thermopsine	486-90-8
HY-N3182	N-Methylnuciferine	754919-24-9
HY-N4317	Ethoxysanguinarine	28342-31-6
HY-N0239	Bulleyaconitine A	107668-79-1
HY-22385	Salsolidine	5784-74-7
HY-B0739	Citicoline	987-78-0
HY-N0166	Gramine	87-52-5
HY-W005963	Methyl 5-hydroxypyridine-2-carboxylate	30766-12-2
HY-17578	Pneumocandin B0	135575-42-7
HY-N0298	Stachydrine	471-87-4

HY-N1282	Seneciphylline	480-81-9
HY-N1933	Allocryptopine	485-91-6
HY-N0069	Solamargine	20311-51-7
HY-N0766	Isorhynchophylline	6859-01-4
HY-N0838	Cephalotaxine	24316-19-6
HY-N2369	Chelidonine	476-32-4
HY-N0252	Catharanthine	2468-21-5
HY-N0901A	Corynoxine B	17391-18-3
HY-N0144	Piperine	94-62-2
HY-N10117	2,3-Bis(3-indolylmethyl) indole	138250-72-3
HY-N7674	Angoline	21080-31-9
HY-W007140	2-Hydroxymethyl-5-hydroxypyridine	40222-77-3
HY-N1100	Vasicinone	486-64-6
HY-100809	Guvacine hydrochloride	6027-91-4
HY-13704	SN-38	86639-52-3
HY-113432	Nudifloramide	701-44-0
HY-N0638	Dendrobine	2115-91-5
HY-101407	Nicotinamide N-oxide	1986-81-8
HY-100806	Kynurenic acid	492-27-3
HY-N0714	Berbamine	478-61-5
HY-107275	Ebeiedinone	25650-68-4
HY-N0300	Tetrahydropalmatine	2934-97-6
HY-N2373A	Palmaturbine (hydroxide)	
HY-13715A	Norepinephrine (hydrochloride)	329-56-6
HY-N6824	Hydroxy-β-sanshool	97465-69-5
HY-Y1129	3-Hydroxypyridine	109-00-2
HY-N0789	Delsoline	509-18-2
HY-N5022	Evolitrine	523-66-0
HY-N0682	Pyridoxine (hydrochloride)	58-56-0
HY-12048	Chelerythrine (chloride)	3895-92-9
HY-N0127	Yohimbine (Hydrochloride)	65-19-0
HY-N0737A	Harmine	442-51-3
HY-W001909	Myosmine	532-12-7
HY-N1584	Halofuginone	55837-20-2
HY-B0739A	Citicoline (sodium)	33818-15-4
HY-123033A	Nicotinamide riboside (chloride)	23111-00-4
HY-N0926	Columbamine	3621-36-1
HY-N3184	N-Methylflindersine	50333-13-6
HY-N0746	Oxysophocarpine	26904-64-3
HY-107271	Imperialine 3-β-D-glucoside	67968-40-5
HY-13768A	Topotecan (Hydrochloride)	119413-54-6
HY-76228	1H-pyrazole	288-13-1
HY-N2361	N-Benzyllinoleamide	18286-71-0
HY-N0217	Benzoylaconine	466-24-0
HY-W001542	5-Hydroxyoxindole	3416-18-0
HY-N0510	Aristolochic acid A	313-67-7
HY-N0221	Daurisoline	70553-76-3
HY-N1483	Guanfu base A	1394-48-5
HY-N2260	Cephaeline (dihydrochloride)	5853-29-2
HY-N0114	Evodiamine	518-17-2
HY-N0300A	Tetrahydropalmatine (hydrochloride)	6024-85-7
HY-N8157	4'-O-Methylpyridoxine	1464-33-1
HY-B0762	Acetyl-L-carnitine (hydrochloride)	5080-50-2
HY-B1021	Vincamine	1617-90-9
HY-N6827	Usaramine N-oxide	117020-54-9

HY-17470	Mizoribine	50924-49-7
HY-N2377	Allosecurinine	884-68-4
HY-N1637	1-Methyl-2-pentyl-4(1H)-quinolinone	22048-98-2
HY-N0902	Dihydrosanguinarine	3606-45-9
HY-N2232	N-Feruloyloctopamine	66648-44-0
HY-N6029	Dehydroevodiamine (hydrochloride)	111664-82-5
HY-N0736	Coptisine (chloride)	6020-18-4
HY-N0674	Dehydrocorydaline	30045-16-0
HY-N0901	Corynoxine	6877-32-3
HY-N0480	Reserpine	50-55-5
HY-N0113	Hordenine	539-15-1
HY-P1940	Maculosin	4549-02-4
HY-101397	Allopurinol riboside	16220-07-8
HY-Y0152	Cinchonine	118-10-5
HY-107811	Harmol	487-03-6
HY-N0414	Trigonelline	535-83-1
HY-N4309A	Lotusine (hydroxide)	3721-76-4
HY-N6637	Senecionine N-oxide	13268-67-2
HY-118341	Clitocine	105798-74-1
HY-16563	Narciclasine	29477-83-6
HY-B0811	Salicylamide	65-45-2
HY-13738A	Raloxifene (hydrochloride)	82640-04-8
HY-N0923	Corydaline	518-69-4
HY-N7625	N-Formylcytisine	53007-06-0
HY-14944	Homoharringtonine	26833-87-4
HY-118824A	N-Feruloylserotonin	68573-23-9
HY-N0750	Monocrotaline	315-22-0
HY-N0175	Cytisinicline	485-35-8
HY-N7612	N-Desmethyl Galanthamine	41303-74-6
HY-N1372A	Fangchinoline	436-77-1
HY-N0759	Acetylcorynoline	18797-80-3
HY-N3033	N-Benzyllinolenamide	883715-18-2
HY-13516	Aloperine	56293-29-9
HY-W009783	1-Deoxymannojirimycin (hydrochloride)	73465-43-7
HY-N0484	Liensinine	2586-96-1
HY-N3536	Canthin-6-one	479-43-6
HY-121936	Yohimbic acid	522-87-2
HY-W007324	Maleimide	541-59-3
HY-N0213	Peiminine	18059-10-4
HY-33037	Phenazine-1-carboxylic acid	2538-68-3
HY-N0663	Talatisamine	20501-56-8
HY-122489	DL-Laudanosine	1699-51-0
HY-N0164	Matrine	519-02-8
HY-W110662	Transtorine	13593-94-7
HY-119674A	Xanthopterin (hydrate)	5979-01-1
HY-12715	Yohimbine	146-48-5
HY-77817	Pyrrole-2-carboxaldehyde	1003-29-8
HY-N0387	Rhynchophylline	76-66-4
HY-N6932	Voacamine	3371-85-5
HY-N2380	N-Benzoyl-(2R,3S)-3-phenylisoserine	132201-33-3
HY-112642	9-Methoxycanthin-6-one	74991-91-6
HY-N0289	Lycorine (hydrochloride)	2188-68-3
HY-B1532	Anabasine	494-52-0
HY-N0282	Colcemid	477-30-5
HY-N2616	Vomicine	125-15-5

HY-N0793	Protopine	130-86-9
HY-N2384A	Febrifugine (dihydrochloride)	32434-42-7
HY-B1178	Cotinine	486-56-6
HY-N0276	Flaconitine	77181-26-1
HY-N0212	Peimine	23496-41-5
HY-N5025	Bullatine A	1354-84-3
HY-113382	N-Methylhydantoin	616-04-6
HY-76705	Methyl N-methylanthranilate	85-91-6
HY-N6011	9-Methoxycamptothecin	39026-92-1
HY-N1089	Vincosamide	23141-27-7
HY-N2080	Songorine	509-24-0
HY-N8728	Aposcopolamine	535-26-2
HY-N2129	N-Nornuciferine	4846-19-9
HY-119529	Jineol	178762-28-2
HY-107383	Tetrahydrobiopterin	17528-72-2
HY-N0226A	Epiberberine (chloride)	889665-86-5
HY-B0459	Scopine	498-45-3
HY-N0837	Veratramine	60-70-8
HY-N0352	Tuberostemonine	6879-01-2
HY-N4188	N-Benzyloctadecanamide	5327-45-7
HY-17577	Berberine (chloride hydrate)	68030-18-2
HY-N2368A	Arecaidine (hydrochloride)	6018-28-6
HY-N2162	12-Epinapelline	110064-71-6
HY-40161	Indole-3-carboxylic acid	771-50-6
HY-N5077	Sinapine	18696-26-9
HY-N1599	16-Epivoacarpine	114027-38-2
HY-N2368	Arecaidine	499-04-7
HY-N0277	Aconine	509-20-6
HY-15758	3,3'-Diindolylmethane	1968-05-4
HY-18569	3-Indoleacetic acid	87-51-4
HY-N1924	Crassicauline A	79592-91-9
HY-13744	Rubitecan	91421-42-0
HY-17568	Nonivamide	2444-46-4
HY-107670	Dihydro-β-erythroidine (hydrobromide)	29734-68-7
HY-N2909	Aurantiamide	58115-31-4
HY-N0110	Palmatine (chloride)	10605-02-4
HY-N2164	3-Deoxyaconitine	3175-95-9
HY-119502	Camalexin	135531-86-1
HY-N3945	Glaucine	475-81-0
HY-W015815	6-Methylnicotinamide	6960-22-1
HY-B0433A	Quinine (hydrochloride dihydrate)	6119-47-7
HY-N0190	Amygdalin	29883-15-6
HY-N6778	Paxilline	57186-25-1
HY-N4030	Humantenmine	82354-38-9
HY-121376	Neoamygdalin	29883-16-7
HY-107276	Yubeinine	157478-01-8
HY-N0147	Rutaecarpine	84-26-4
HY-N7061	Tropine	120-29-6
HY-N6972	Cepharanthine	481-49-2
HY-N2307A	Lirinidine	54383-28-7
HY-N2063	12-Ethyl-9-hydroxycamptothecin	119577-28-5
HY-N0471	L-Hyoscyamine	101-31-5
HY-N4238	Dehydrocorydaline (nitrate)	13005-09-9
HY-N0224	Epigoitrin	1072-93-1
HY-N0110B	Palmatine (hydroxide)	131-04-4

HY-N2411	Geissoschizine methyl ether	60314-89-8
HY-W018601	Nortropine	538-09-0
HY-107339	Deserpidine	131-01-1
HY-W011151	trans-Zeatinriboside	6025-53-2
HY-12882A	Ifenprodil (tartrate)	23210-58-4
HY-N9251	Cyclo(Ile-Ala)	90821-99-1
HY-N0114A	(±)-Evodiamine	518-18-3
HY-12710A	Rauwolscine (hydrochloride)	6211-32-1
HY-W008350	(+)-Sparteine	492-08-0
HY-N0931	Santacruzamate A	1477949-42-0
HY-N2108	7-Ethylcamptothecin	78287-27-1
HY-N7608	Beiwutine	76918-93-9
HY-N0446	10-Methoxycamptothecin	19685-10-0
HY-N2060	Evocarpine	15266-38-3
HY-N1198	Strictosamide	23141-25-5
HY-N5079	Lotaustralin	1973415-50-7
HY-N0218	Benzoylmesaconine	63238-67-5
HY-W007376	Indole-3-carboxaldehyde	487-89-8
HY-N7010	(-)-Corynoxidine	57906-85-1
HY-N0687	Vindoline	2182-14-1
HY-N0925	Tetrahydroberberine	522-97-4
HY-N3196	Neotuberostemonine	143120-46-1
HY-W098556	4-Hydroxyhygric acid	4252-82-8
HY-113236	p-Synephrine	614-35-7
HY-N2309	Kainic acid	487-79-6
HY-N0535	(+)-Magnoflorine (chloride)	6681-18-1
HY-N2393	Kukoamine B	164991-67-7
HY-N0488	Vincristine (sulfate)	2068-78-2
HY-N0404	Sinigrin	3952-98-5
HY-N0415	Trigonelline (chloride)	6138-41-6
HY-40135	L-Hydroxyproline,BioReagent, suitable for cell culture	51-35-4
HY-13417A	AICAR (phosphate)	681006-28-0
HY-W012683	Iminodiacetic acid	142-73-4
HY-136648A	2'-Deoxyadenosine-5'-triphosphate (trisodium)	54680-12-5
HY-W104368	Nicotinic acid riboside	17720-18-2
HY-14860	1-Deoxynojirimycin	19130-96-2
HY-N1914	Ergothioneine	497-30-3
HY-A0129	Histamine (phosphate)	51-74-1
HY-N0749A	Jatrorrhizine (hydroxide)	483-43-2
HY-N0924	(±)-Stylopine	4312-32-7
HY-N2560	Senecionine	130-01-8
HY-13764	Tetrandrine	518-34-3
HY-N1436	L-(+)-Abrine	526-31-8
HY-N0052A	Sanguinarine (chloride)	5578-73-4
HY-N2005	Cycleanine	518-94-5
HY-N0068	Solasodine	126-17-0
HY-N2365	N-Benzylpalmitamide	74058-71-2
HY-N0836	Jervine	469-59-0
HY-N6894A	Pseudocoptisine (chloride)	30044-78-1
HY-N0430A	Coptisine (Sulfate)	1198398-71-8
HY-N0741	Leonurine	24697-74-3
HY-N6865	Groenlandicine	38691-95-1
HY-N0590	Corynoxeine	630-94-4

HY-N2079	(-)-Securinine	5610-40-2
HY-N8346	8-Oxocoptisine	19716-61-1
HY-N0498	Nitidine (chloride)	13063-04-2
HY-N0724	Mesaconitine	2752-64-9
HY-N0592A	Demethyleneberberine (chloride)	16705-03-6
HY-N0935	Ligustrazine (hydrochloride)	76494-51-4
HY-N5014	Liensinine (perchlorate)	2385-63-9
HY-N6931	Usaramine	15503-87-4
HY-N7401	Entadamide-A-β-D-glucopyranoside	138916-58-2
HY-N0158	Oxymatrine	16837-52-8
HY-N2021A	Phosphoramidon (Disodium)	164204-38-0
HY-N0388	Gelsemine	509-15-9
HY-N8157	4'-O-Methylpyridoxine	1464-33-1
HY-N0107	Cyclovirobuxine D	860-79-7
